# Arrangement of water molecules and high proton conductivity of tunnel structure phosphates, KMg_1−*x*_H_2*x*_(PO_3_)_3_·*y*H_2_O†

**DOI:** 10.1039/d0ra00690d

**Published:** 2020-02-24

**Authors:** Yasuaki Matsuda, Kousei Funakoshi, Ryosuke Sebe, Genki Kobayashi, Masao Yonemura, Nobuyuki Imanishi, Daisuke Mori, Shinya Higashimoto

**Affiliations:** Department of Applied Chemistry, Faculty of Engineering, Osaka Institute of Technology 5-16-1 Ohmiya Asahi-Ku Osaka 535-8585 Japan yasuaki.matsuda@oit.ac.jp; Research Center of Integrative Molecular Systems (CIMoS), Institute for Molecular Science 38 Nishigonaka, Myodaiji Okazaki Aichi 444-8585 Japan; Institute of Materials Structure Science, High Energy Accelerator Research Organization 1-1 Oho Tsukuba Ibaraki 305-0801 Japan; Department of Chemistry for Materials, Graduate School of Engineering, Mie University 1577 Kurimamachiya-cho Tsu Mie 514-8507 Japan

## Abstract

A fast proton conductor was investigated in a mixed-valence system of phosphates with a combination of large cations (K^+^) and small cations (Mg^2+^), which resulted in a new phase with a tunnel structure suitable for proton conduction. KMg_1−*x*_H_2*x*_(PO_3_)_3_·*y*H_2_O was synthesized by a coprecipitation method. A solid solution formed in the range of *x* = 0–0.18 in KMg_1−*x*_H_2*x*_(PO_3_)_3_·*y*H_2_O. The structure of the new proton conductor was determined using neutron and X-ray diffraction measurements. KMg_1−*x*_H_2*x*_(PO_3_)_3_·*y*H_2_O has a tunnel framework composed of face-shared (KO_6_) and (MgO_6_) chains, and PO_4_ tetrahedral chains along the *c*-direction by corner-sharing. Two oxygen sites of water molecules were detected in the one-dimensional tunnel, one of which exists as a coordination water of K^+^ sites. Multi-step dehydration was observed at 30 °C and 150 °C from thermogravimetric/differential thermal analysis measurements, which reflects the different coordination environments of the water of crystallization. Water molecules are connected to PO_4_ tetrahedra by hydrogen bonds and form a chain along the *c*-axis in the tunnel, which would provide an environment for fast proton conduction associated with water molecules. The KMg_1−*x*_H_2*x*_(PO_3_)_3_·*y*H_2_O sample with *x* = 0.18 exhibited high proton conductivity of 4.5 × 10^−3^ S cm^−1^ at 150 °C and 7.0 × 10^−3^ S cm^−1^ at 200 °C in a dry Ar gas flow and maintained the total conductivity above 10^−3^ S cm^−1^ for 60 h at 150 °C under N_2_ gas atmosphere.

## Abstract 2

A fast proton conductor exhibiting high proton conductivity of 7.0 × 10^−3^ S cm^−1^ at 200 °C in a dry Ar gas flow was developed by designing water chains in a rigid tunnel framework.

## Graphical abstract

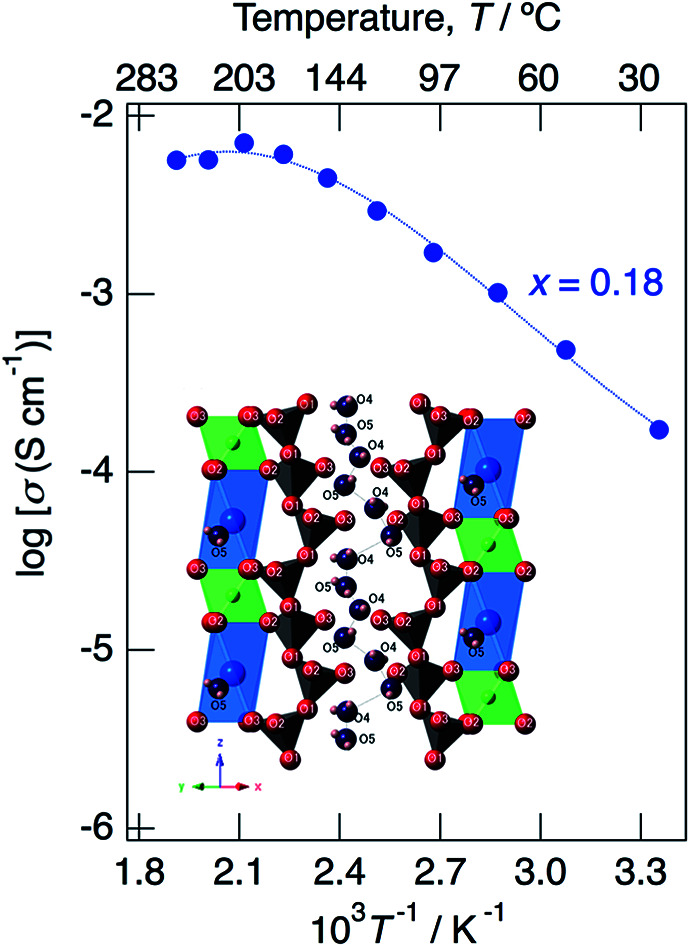

## Introduction

Proton conduction is an essential phenomenon common in the metabolism of biological systems^1–3^ and the operation of electrochemical devices such as fuel cells.^4–9^ An understanding of proton conduction and the development of fast proton conductors are important for material science, chemistry, and biology due to fundamental interests and potential applications. Fast proton conduction in solids has been achieved by the design of proton conduction pathways in their crystal structures. The discovery and structural investigation of new proton conductors provide deep insights into the origin of fast proton conduction in solids and a means to design better proton conductors. Studies based on representative proton conductors^10–16^ have promoted the development of fuel cell electrolyte materials.^7–9^ Incombustible inorganic solids have a potential to extend the operation temperature of proton solid electrolytes, however the design of materials that exhibit high proton conductivity and thermal stability is still a big challenge because of the difficulty controlling hydrogen bonds in the solids.

Since the framework formed by hydrogen bonds is difficult to improve thermal stability, we have investigated materials that have a fast proton diffusion pathway in the rigid framework formed by covalent and ionic bonds. Among the inorganic solids, we have focused on mixed-valence systems of phosphates because they tend to form open frameworks by the connection of PO_4_ tetrahedra. Furthermore, mixed-valence systems may introduce excess protons into the structure by changing the cation molar ratio, and these protons form hydrogen bonds with PO_4_ tetrahedra. These protons result in changes to the hydrogen bond network that is suitable for fast proton conduction in the host structure. Based on this concept, we have discovered a tunnel phosphate, RbMg_1−*x*_H_2*x*_(PO_3_)_3_·*y*H_2_O, which exhibits high proton conductivity above 10^−3^ S cm^−1^ over a wide temperature range from room temperature to 250 °C.^17^ The framework of RbMg_1−*x*_H_2*x*_(PO_3_)_3_·*y*H_2_O is composed of the corner-sharing of face-shared (RbO_6_) and (MgO_6_) chains and corner-shared PO_4_ tetrahedral chains. Excess protons introduced by the introduction of vacancies at Mg^2+^ sites induce hydrophilic head groups (–PO_4_H) into the PO_4_ framework, which act as binding sites for water molecules. Therefore, water molecules are distributed adjacent to each other and form chains along the PO_4_ tetrahedral chains in the one-dimensional tunnel. This provides an environment for fast proton diffusion, which is similar to the proton channels observed in biological systems.^17^

The tunnel phosphate is attractive as a proton solid electrolyte if it can be synthesized with inexpensive elements and maintain high proton conductivity over 10^−3^ S cm^−1^ above 100 °C at which most of proton conductors containing water molecules become unstable. However, the relationship between crystal structure and the combination of constitute cations in the water containing phosphates with alkali and alkaline earth cations have not been clarified yet. Therefore, except for RbMg_1−*x*_H_2*x*_(PO_3_)_3_·*y*H_2_O, proton conductors with the water chain in the tunnel framework haven't been discovered. To obtain new proton conductor, we attempted to synthesize a new tunnel phosphate combining Mg^2+^ and K^+^ by a coprecipitation method that is suitable for low-temperature synthesis. The replacement of Rb^+^ for a smaller alkali cation of K^+^ would cause shrinkage of the tunnel framework. We expected the improvement of the proton conductivity by decreasing the distance between water molecules in the tunnel due to lattice contraction. To understand the relationship between the arrangement of water molecules and proton conductivity, the crystal structure of the new proton conductor was determined using neutron and X-ray diffraction (XRD) measurements. Excess protons were introduced by vacancies at Mg^2+^ sites to improve proton conductivity. In present study, the synthesis, crystal structure and proton conductivity of KMg_1−*x*_H_2*x*_(PO_3_)_3_·*y*H_2_O are discussed.

## Experimental

The KMg_1−*x*_H_2*x*_(PO_3_)_3_·*y*H_2_O series was synthesized by a coprecipitation method. Appropriate molar ratios of K_2_CO_3_ (Nacalai Tesque, >99.5%) and (MgCO_3_)_4_Mg(OH)_2_·*x*H_2_O (Nacalai Tesque) were weighed and dissolved in 30 mL of phosphoric acid solution (0.5 mol L^−1^, Kanto Chemical Lab. Co.). To compensate for the compositional deviation of the starting materials, a 2 mol% excess of potassium was added. The solution was heated at 120 °C in air for several days and a white colored residual powder was obtained. The sample powder obtained was pressed into a pellet (10 mm diameter, 1–2 mm thick) and then heated in air at 250 °C for 12–24 h on a polytetrafluoroethylene (PTFE) sheet in a watch glass with an intermediate grinding step. XRD patterns of the powder samples were obtained using a diffractometer (Rigaku RINT2000) with Cu Kα radiation in the 2*θ* range from 10 to 90° at 0.02° step widths. Thermogravimetry/differential thermal analysis (TG/DTA; Rigaku Thermo Plus EVO TG 8120) were performed at a heating rate of 5 °C min^−1^ in a N_2_ gas flow. The structures of the samples were refined by X-ray Rietveld analysis using the RIETAN-FP program.^18^ Neutron diffraction (ND) data were collected using a time-of-flight (TOF) neutron powder diffractometer (SPICA at J-PARC, Japan). Samples (*ca*. 1.0 g) were placed in cylindrical vanadium cells (12 mm diameter, 55 mm height). The structural parameters were refined by Rietveld analysis using the Z-Rietveld program.^19^ The ionic conductivities were measured by the AC impedance method in the temperature range from room temperature to 250 °C in a dry Ar gas flow and under N_2_ gas atmosphere over the frequency range of 10 Hz to 1 MHz using a frequency response analyzer (Bio-Logic VSP 300) and an LCR meter (Hioki MI3536). The moisture in the Ar gas was removed with a gas purifier to less than 0.03%. Measurements were performed using a sample pellet (10 mm diameter, 1–2 mm thick) with gold electrodes on each side. The relative density of the measured pellet was approximately 80%.

## Results and discussion

**Fig. 1:**
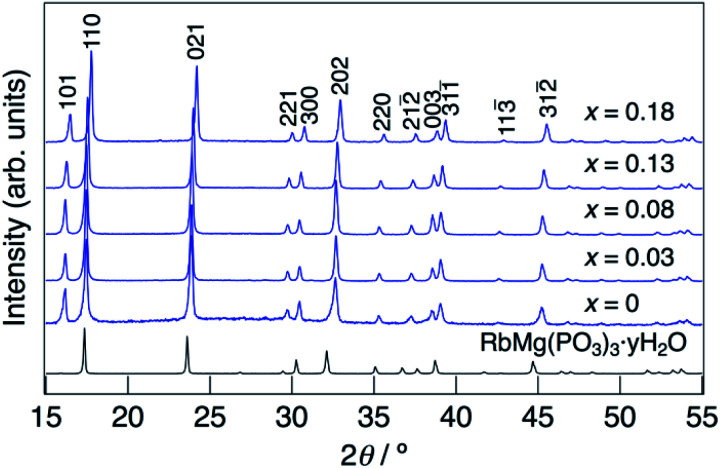
XRD patterns for KMg_1−*x*_H_2*x*_(PO_3_)_3_·*y*H_2_O synthesized in an air at 250 °C for 12 h.

XRD patterns for KMg_1−*x*_H_2*x*_(PO_3_)_3_·*y*H_2_O synthesized in air at 250 °C are shown in Fig. 1. The diffraction patterns for all samples are similar to that of RbMg(PO_3_)_3_·*y*H_2_O,^17^ and the diffraction peaks were indexed to a rhombohedral unit cell. In contrast to KMg(PO_3_)_3_ with the layered structure reported previously,^20^ the new phase with a tunnel structure was most likely obtained due to the low temperature of synthesis by the coprecipitation method. Above the composition of *x* = 0.18, crystalline samples were not obtained by heating at 250 °C. The diffraction peaks of KMg_1−*x*_H_2*x*_(PO_3_)_3_·*y*H_2_O were observed at higher 2*θ* angles than those of RbMg(PO_3_)_3_·*y*H_2_O, which is due to the replacement of Rb^+^ with the smaller K^+^ cation. The diffraction peaks tended to shift to higher angles with an increase in *x*, which indicates the formation of a solid solution.

**Fig. 2:**
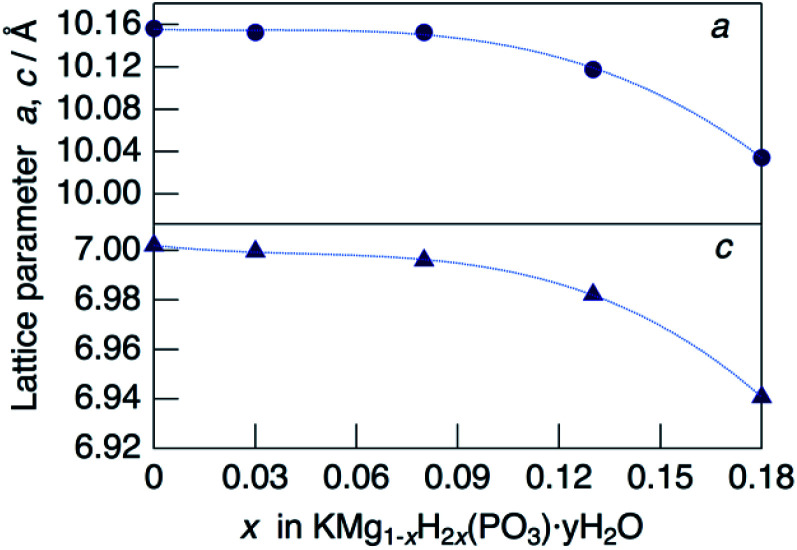
Lattice parameters as a function of *x* in KMg_1−*x*_H_2*x*_(PO_3_)_3_·*y*H_2_O.

Fig. 2 shows the change in the lattice parameter as a function of *x* in KMg_1−*x*_H_2*x*_(PO_3_)_3_·*y*H_2_O. The lattice parameter *a* remained constant, while *c* decreased slightly from *x* = 0 to 0.08, after which both *a* and *c* decreased largely from *x* = 0.08 to *x* = 0.18. Several factors may affect the change in the lattice parameters of the solid solution, such as vacancies at Mg^2+^ sites, excess protons and the water of crystallization. A similar tendency was observed for RbMg_1−*x*_H_2*x*_(PO_3_)_3_H_2_O.^17^

**Fig. 3:**
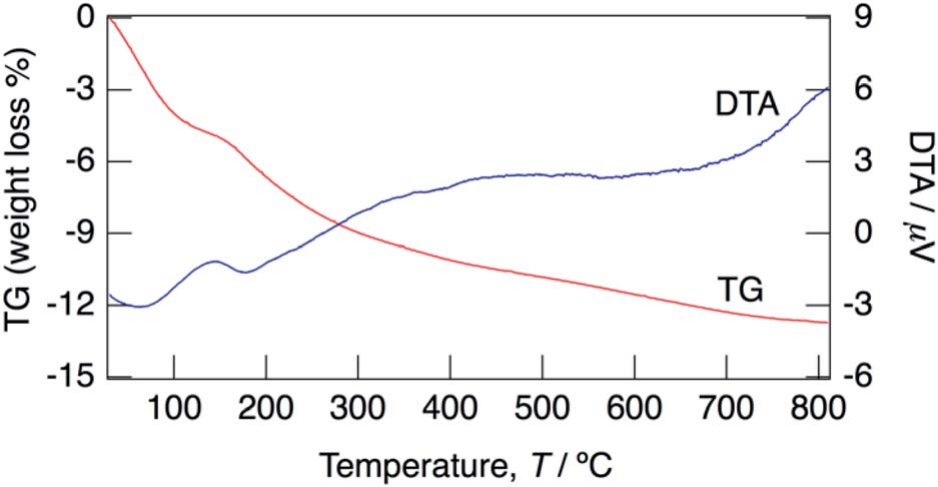
TG/DTA curves for KMg_1−*x*_H_2*x*_(PO_3_)_3_·*y*H_2_O with *x* = 0.18 measured at a heating rate of 5 °C min^−1^ in a N_2_ gas flow.

Fig. 3 shows TG/DTA curves for the sample with *x* = 0.18 between 30 and 800 °C. Water loss proceeded from 30 °C and at 150 °C. At the initial step, a large weight loss was observed at 30–100 °C, and then a small amount of water loss occurred at 100–150 °C. It is considered that the initial step corresponds to the loss of adsorbed water and water of crystallization, and the second step corresponds to the loss of only water of crystallization. The water content calculated based on the occupancy values of oxygen of water molecules obtained by structural refinement mentioned later suggests the loss of water of crystallization is the predominant contributor to the weight loss at the initial step. It is expected that the weight loss at 30–100 °C includes a small amount of adsorbed water, however clear separation of weight loss corresponding to desorption of water of crystallization was difficult from the TG/DTA curves. The water contents of KMg_1−*x*_H_2*x*_(PO_3_)_3_·*y*H_2_O with *x* = 0.18 calculated from the weight loss in the range of 30–150 °C as the initial dehydration step, and that in 150–800 °C as the second step were 0.8H_2_O and 1.1H_2_O per unit formula.

**Fig. 4:**
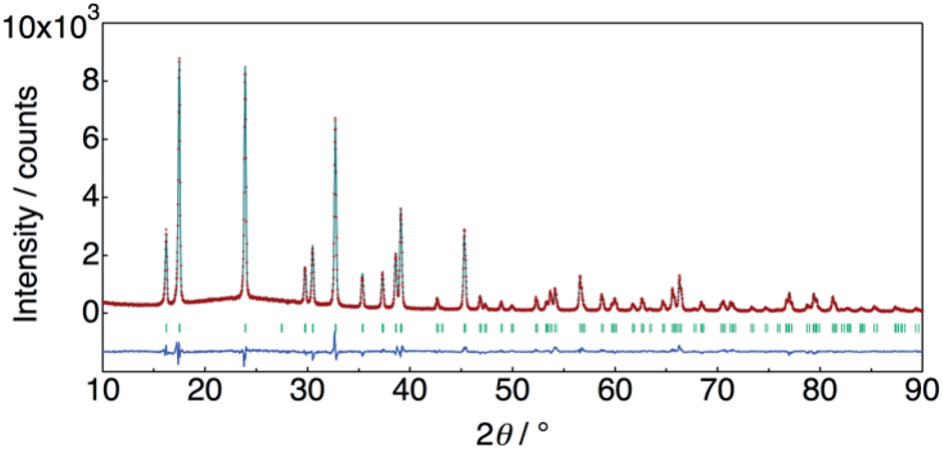
X-ray Rietveld refinement pattern for KMg_0.82_H_0.36_(PO_3_)_3_·1.9H_2_O.

**Fig. 5:**
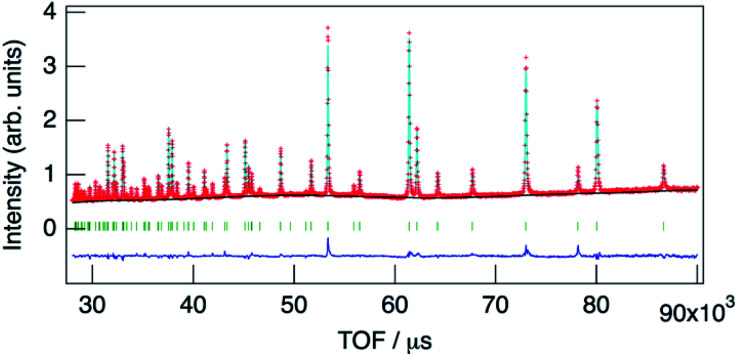
Neutron Rietveld refinement pattern for KMg_0.82_H_0.36_(PO_3_)_3_·1.9H_2_O.

The structure of KMg_1−*x*_H_2*x*_(PO_3_)_3_·*y*H_2_O was determined by Rietveld refinement using XRD and ND data. Two structural models, RbMg(PO_3_)_3_·*y*H_2_O: space group *R*32 with Mg in 3a, K in 3b, P in 9e, O(1) in 18f, O(2) in 9d and O(3) in 18f sites,^17^ and KNi(PO_3_)_3_: space group *R*3 with Mg in 3a, K in 3a, P in 9b, O(1) in 9b, O(2) in 9b and O(3) in 9b sites^21^ were examined as initial models for X-ray Rietveld refinement. The refinement based on the former model resulted in high values of the conventional agreement factors. The difference Fourier map showed residuals at K and Mg sites, which suggested the irrelevant atomic positions of these cations. Therefore, the refinement based on the latter model with low symmetry was examined to determine their atomic positions. As a result, reliability factors of *R*_wp_ = 8.31%, *R*_p_ = 6.40%, *R*_e_ = 5.17%, *R*_B_ = 3.81%, and *R*_F_ = 2.38% with refined lattice parameters of *a* = 10.1594(3) Å and *c* = 6.9986(2) Å were obtained. Refinement provided an occupancy value of 0.824(5) for magnesium at the 3a site, which was consistent with the nominal composition. Two oxygen sites corresponding to the water of crystallization were newly determined. ND data were refined based on the result using the structural model with space group *R*3. Proton sites assumed by the difference Fourier map were refined. The final XRD and ND Rietveld refinement patterns for KMg_0.82_H_0.36_(PO_3_)_3_·1.9H_2_O are shown in Fig. 4 and 5, respectively. The refined structural parameters are listed in Table 1, and the selected bond lengths and bond angles are summarized in Table S1.†

**Table 1:** Refined structural parameters for KMg_0.82_H_0.36_(PO_3_)_3_·1.9H_2_O^a^

Atom	Site	*g*	*x*	*y*	*z*	*B*/Å^2^
K	3a	1.0	0	0	0.4907(5)	0.5
Mg	3a	0.824	0	0	0	0.5
P	9b	1.0	0.013(10)	0.5681(10)	0.4648(4)	0.5
O(1)	9b	1.0	0.0228(10)	0.6344(10)	0.2547(4)	1.0
O(2)	9b	1.0	0.0827(10)	0.1915(13)	0.8234(4)	1.0
O(3)	9b	1.0	0.1866(10)	0.1058(10)	0.1716(4)	1.0
O(4)	9b	0.219(2)	0.4039(4)	−0.005(3)	0.1880(5)	1.2
O(5)	9b	0.336(3)	0.5062(3)	0.5903(3)	0.0056(5)	1.2
H(1)	9b	0.238(4)	0.0552(11)	0.3219(9)	0.6138(12)	1.6
H(2)	9b	0.372(3)	0.4705(8)	0.0171(6)	0.2468(8)	1.6
H(3)	9b	0.355(4)	0.4901(7)	0.5350(6)	0.0932(8)	1.6
H(4)	9b	0.190(4)	0.5138(2)	0.6021(13)	0.014(17)	1.6

a Space group: *R*3 (146), *a* = 10.1726(10) Å, *c* = 7.008(10) Å, *S*^2^: 7.12, *R*_wp_ (%): 2.48, *R*_p_ (%): 1.84, *R*_e_ (%): 0.93, *R*_B_ (%): 4.73, *R*_F_ (%): 2.66.

**Fig. 6:**
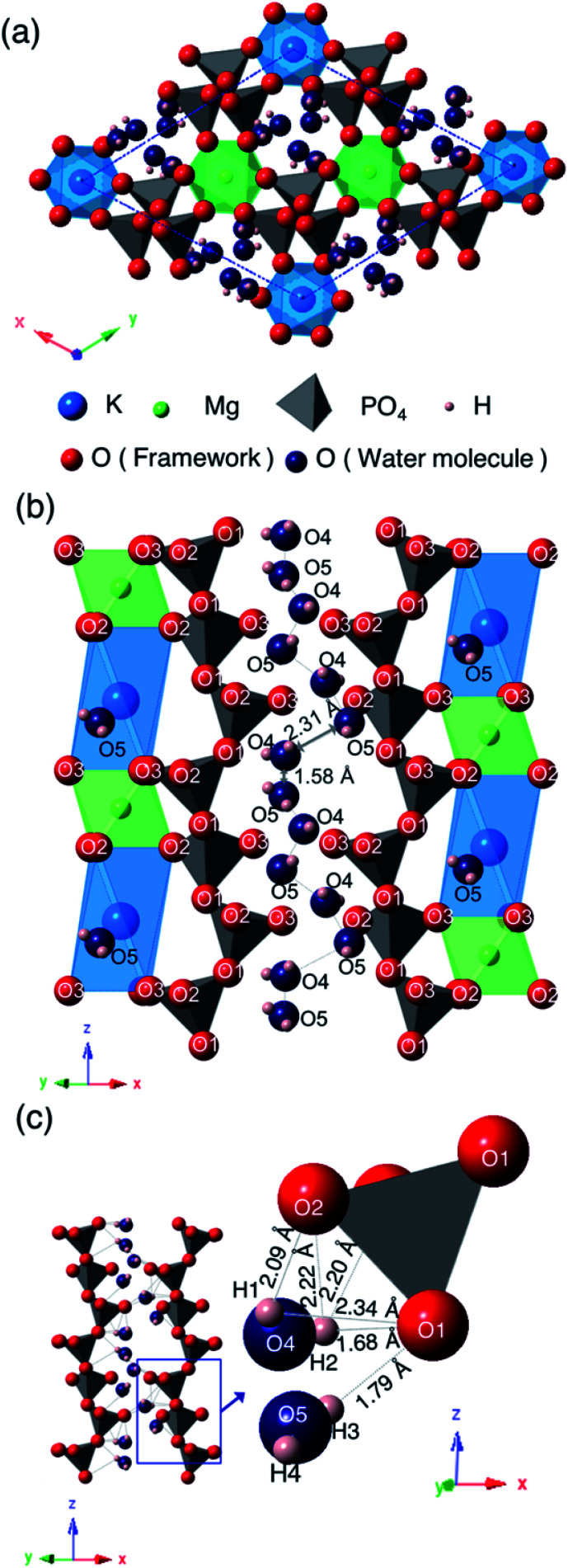
Crystal structure of KMg_0.82_H_0.36_(PO_3_)_3_·1.9H_2_O viewed along the [001] direction (a), framework structure viewed along [110] direction (b), and one-dimensional water chain and the local environment of water molecules located in the one-dimensional tunnel formed by PO_4_ tetrahedral chains (c).

Fig. 6 shows the crystal structure of KMg_0.82_H_0.36_(PO_3_)_3_·1.9H_2_O viewed along the [001] direction (a), the framework structure viewed along the [110] direction (b), and a one-dimensional water chain with the local environment of water molecules located in the one-dimensional tunnel formed by PO_4_ tetrahedra (c). The PO_4_ tetrahedra are slightly distorted from the ideal tetrahedron; structural refinement results indicated that the P–O bond lengths and O–P–O bond angles are varied in the ranges of 1.38–1.71 Å and 97.2–124°, which are slightly deviated from the sum of the ionic radii (1.55 Å) and the ideal bond angle of a tetrahedron (109.5°). The PO_4_ tetrahedra are connected with each other by corner-sharing at O(1) to form chains along the *c*-axis direction. The bond lengths between K^+^ and oxygen are varied at 2.78–2.88 Å and those between Mg^2+^ and oxygen are 2.04–2.10 Å. K^+^ is situated at the off-centered position at the octahedral sites. KO_6_ octahedra and MgO_6_ octahedra are ordered and form chains along the *c*-axis direction by face-sharing. These chains are connected with PO_4_ tetrahedra by corner-sharing at O(2) and O(3). Water molecules are located in the one-dimensional tunnel along PO_4_ tetrahedral chains in the *c*-axis direction.

Two oxygen, O(4) and O(5), and four hydrogen sites, H(1), H(2), H(3) and H(4), exist in the one-dimensional tunnel and they form two water molecules of H(1)–O(4)–H(2) and H(3)–O(5)–H(4). Bond distances and angles between the oxygen and proton sites are as follows: O(4)–H(1): 0.28 Å; O(4)–H(2): 0.60 Å; O(5)–H(3): 0.57 Å; O(5)–H(4): 0.30 Å; H(1)–O(4)–H(2): 118°; H(3)–O(5)–H(4): 137°. These bond distances are comparable to the O–H bonds of water molecules (<1.0 Å), which suggests that these bonds correspond to the covalent bonds of water molecules. Bond angles of 118° and 137° for H(1)–O(4)–H(2) and H(3)–O(5)–H(4), respectively, are slightly distorted from the ideal bond angle of 105° in H–O–H for a water molecule. The occupancy values of O(4) and O(5) are 0.22 and 0.34, respectively. The sum of water content calculated from these occupancy values is 1.7H_2_O per unit formula, which is comparable to the value of 1.9H_2_O per unit formula calculated from the weight loss from 30 to 800 °C by TG/DTA measurement. The small difference between these may be due to desorption of adsorbed water during the initial weight loss step.

The water molecules are connected to PO_4_ tetrahedral chains through the hydrogen bonds. Both of the H(2)–O(1) and H(3)–O(1) bonds with respective distances of 1.68 and 1.79 Å, which are considerably shorter than the hydrogen bond distances in water-contained materials,^22,23^ correspond to values between covalent bonding and hydrogen bonding. Large occupancy values of H(2) and H(3) suggest the coexistence of protons of water molecules and excess protons introduced by vacancies at Mg^2+^ sites. Intermediate H(2)–O(1) and H(3)–O(1) bond distances, and the slightly long covalent bonds of O(4)–H(2) and O(5)–H(3) may reflect the existence of excess protons between water molecules and the PO_4_ tetrahedral framework. Oxygen of water molecules in the tunnel are located at sites near to KO_6_ octahedra, which may be due to the preference of large cations for a large coordination number of anions.

The distances of K–O(4) and K–O(5) are 3.11 Å and 2.36 Å, respectively. The short K–O(5) distance indicates that O(5) and K are tightly connected and O(5) must be an oxygen of the coordination water of K. The water content calculated from the occupancy value of O(4) is almost the same as that calculated from the weight loss at the initial step in the TG curve, and that calculated from the occupancy value of O(5) is in good agreement with that calculated from the weight loss at the second dehydration step. The multi-step dehydration observed by thermal measurement is thus due to the different coordination environments of water molecules.

Two water molecules are ordered along the PO_4_ tetrahedral chains in the one-dimensional tunnel and form chains in the *c*-direction. Adjacent water molecules are situated in the short distance of 1.58 Å and 2.31 Å for O(4)–O(5), which is too short for oxygen to occupy these sites simultaneously. These two oxygen sites are thus partially occupied with occupation parameters of 0.22 and 0.34, respectively, which are reasonable when considering the partial distribution of water molecules over these sites.

**Fig. 7:**
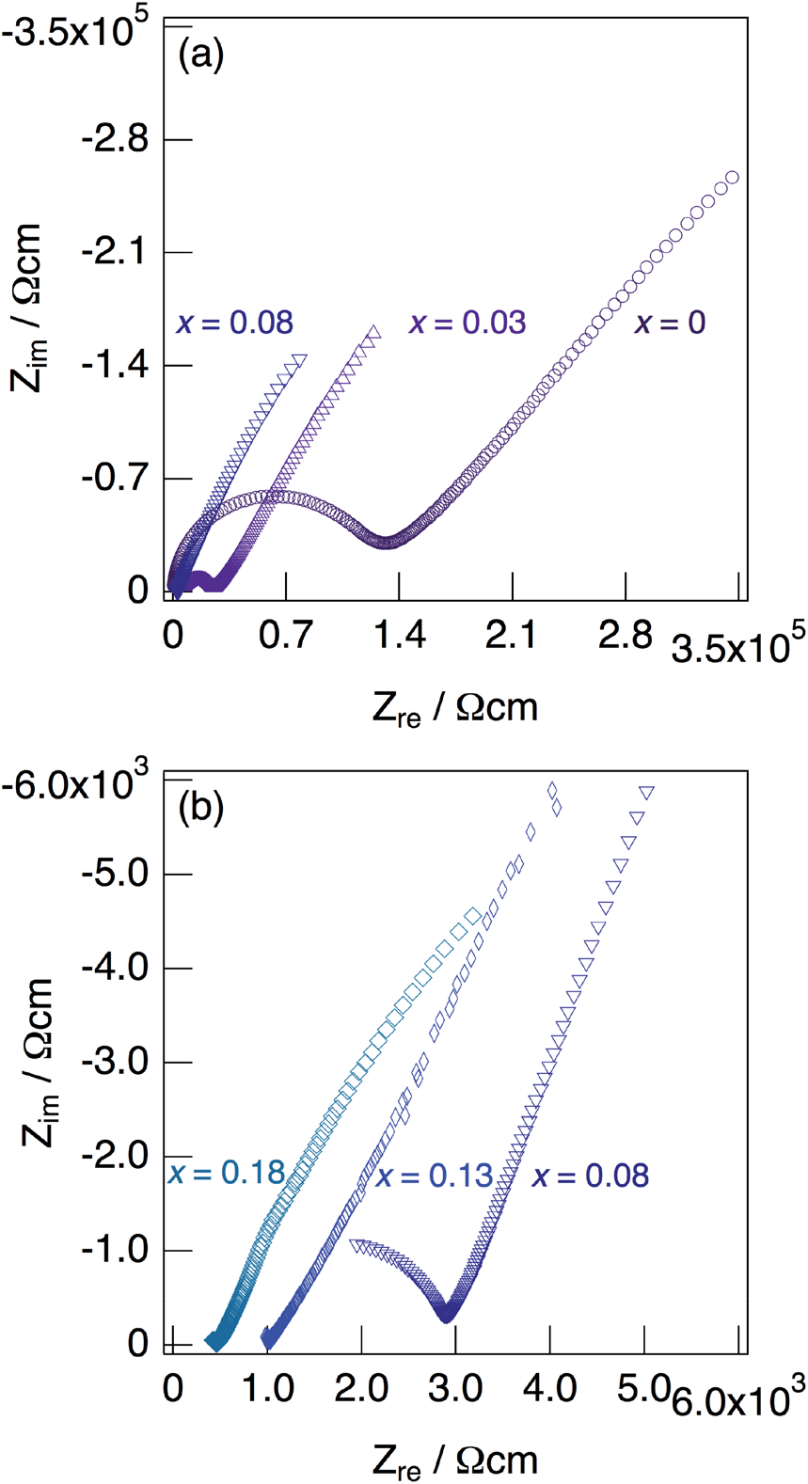
Impedance plots for KMg_1−*x*_H_2*x*_(PO_3_)_3_·*y*H_2_O with *x* = 0–0.08 (a) and *x* = 0.08–0.18 (b) measured under N_2_ gas atmosphere in the closed cell at 150 °C.

**Fig. 8:**
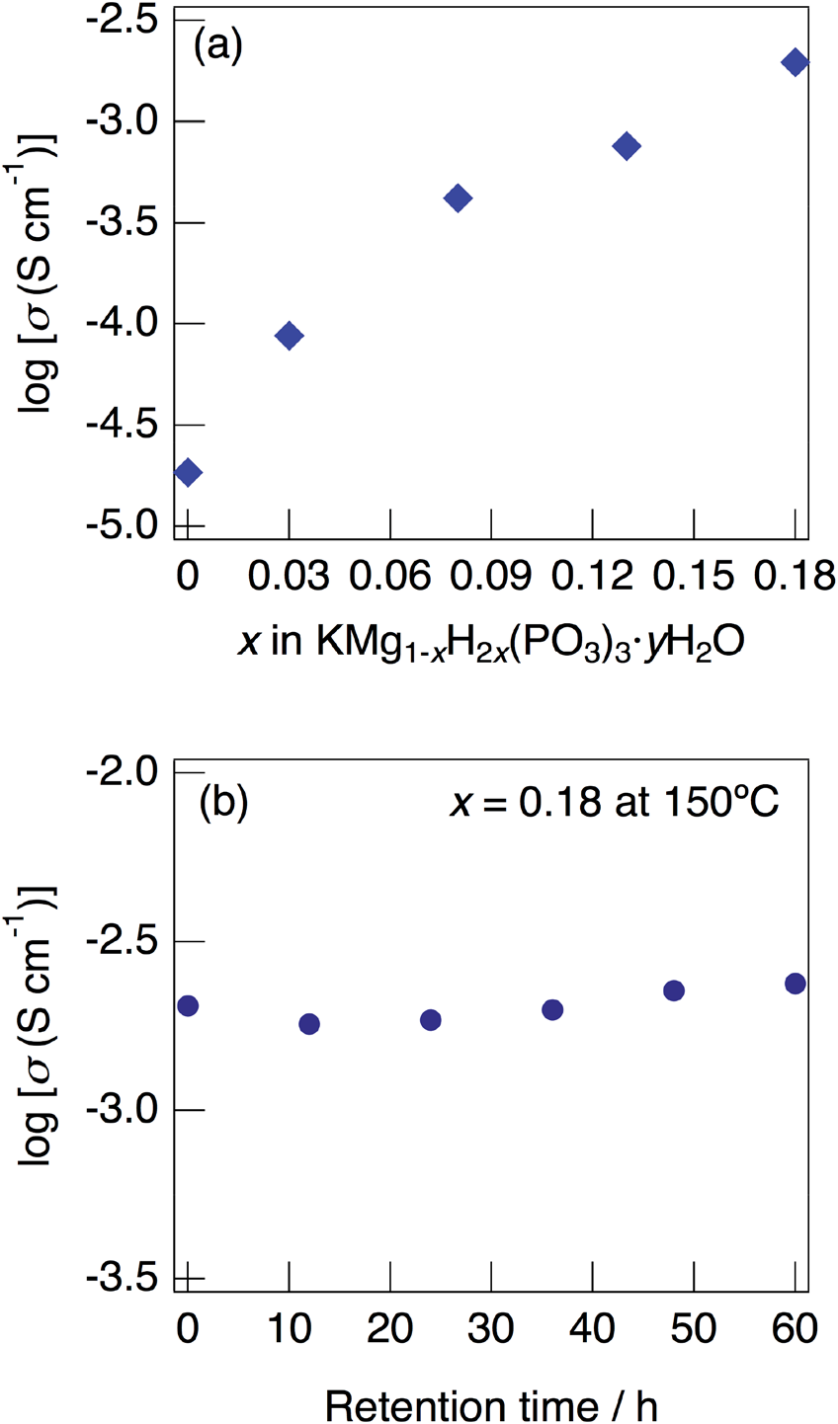
Variation of total conductivity as a function of *x* in KMg_1−*x*_H_2*x*_(PO_3_)_3_·*y*H_2_O (a), and time dependence of the total conductivity of the sample with *x* = 0.18 (b) measured at 150 °C under N_2_ gas atmosphere in the closed cell.

Fig. 7 shows impedance plots for KMg_1−*x*_H_2*x*_(PO_3_)_3_·*y*H_2_O with *x* = 0–0.08 (a) and *x* = 0.08–0.18 (b) measured at 150 °C under N_2_ gas atmosphere in the closed cell. Fig. 8 shows variation of total conductivity as a function of *x* in KMg_1−*x*_H_2*x*_(PO_3_)_3_·*y*H_2_O (a), and time dependence of the total conductivity of the sample with *x* = 0.18 (b) measured at 150 °C under N_2_ gas atmosphere in the closed cell. The vapor pressure in the cell should be saturated considering the amount of dehydration observed in the TG curve and the volume of the space in the cell. The impedance plots measured at 150 °C showed a part of semicircle due to the grain boundary and a spike due to the electrode contribution. The total resistivity was obtained from the real axis intercepts of the semicircle corresponding to the grain boundary at the low frequency side. The total conductivity increased with increasing *x* in KMg_1−*x*_H_2*x*_(PO_3_)_3_·*y*H_2_O. The introduction of excess protons enhances proton conductivity.

The sample with *x* = 0.18 maintained a high total conductivity above 10^−3^ S cm^−1^ at 150 °C and exhibited the total conductivity of 2.4 × 10^−3^ S cm^−1^ at 60 h. This material maintains high-ionic conductivity in the measurement atmosphere in which water of crystallization is retained.

**Fig. 9:**
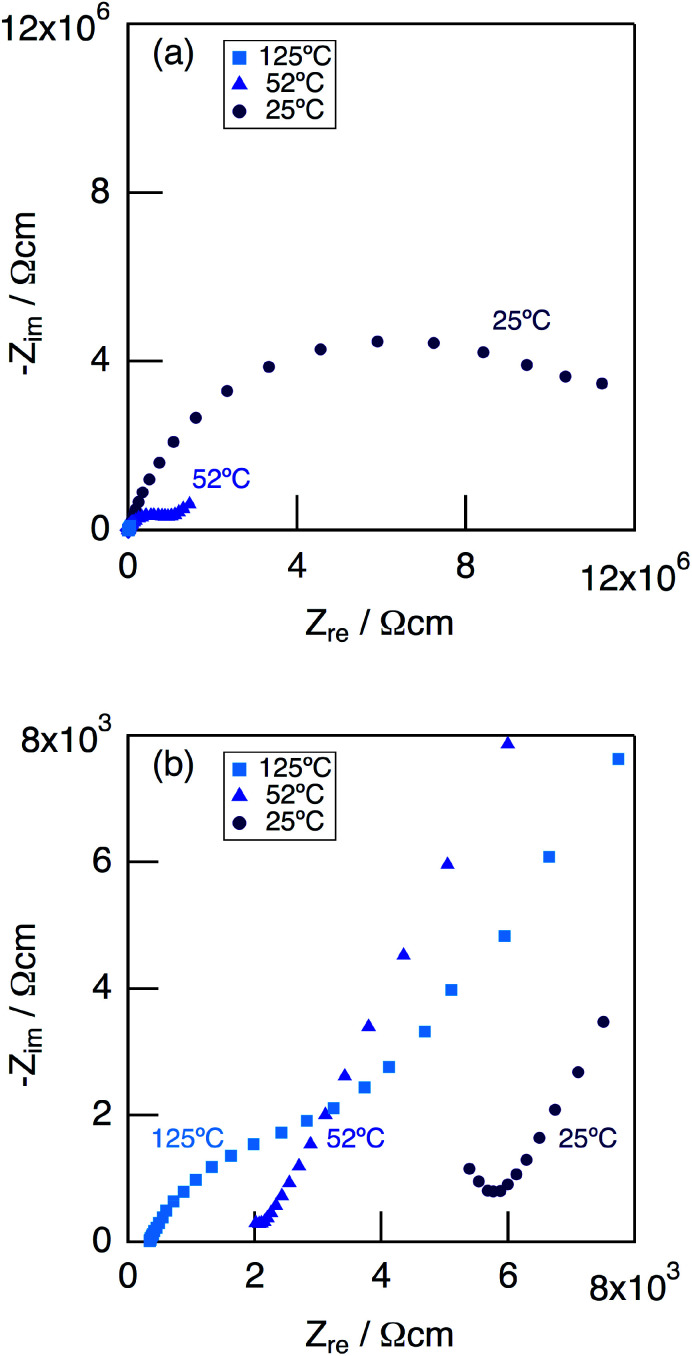
Impedance plots for KMg_1−*x*_H_2*x*_(PO_3_)_3_·*y*H_2_O with *x* = 0.18 measured in a dry Ar gas flow at 25–125 °C (a), and expansion of the high frequency part (b).

Fig. 9 shows impedance plots for KMg_1−*x*_H_2*x*_(PO_3_)_3_·*y*H_2_O with *x* = 0.18 measured under dry Ar gas flow at 25–125 °C (a), and expansion of the high frequency part (b). At 25 °C, a part of the semicircle corresponding to the bulk and a large semicircle due to the grain boundary were observed. At 52 °C, an end part of the semicircle due to the bulk and a second semicircle due to the grain boundary were observed in the high frequency range, while a spike due to the electrode contribution in the low-frequency range.

**Fig. 10:**
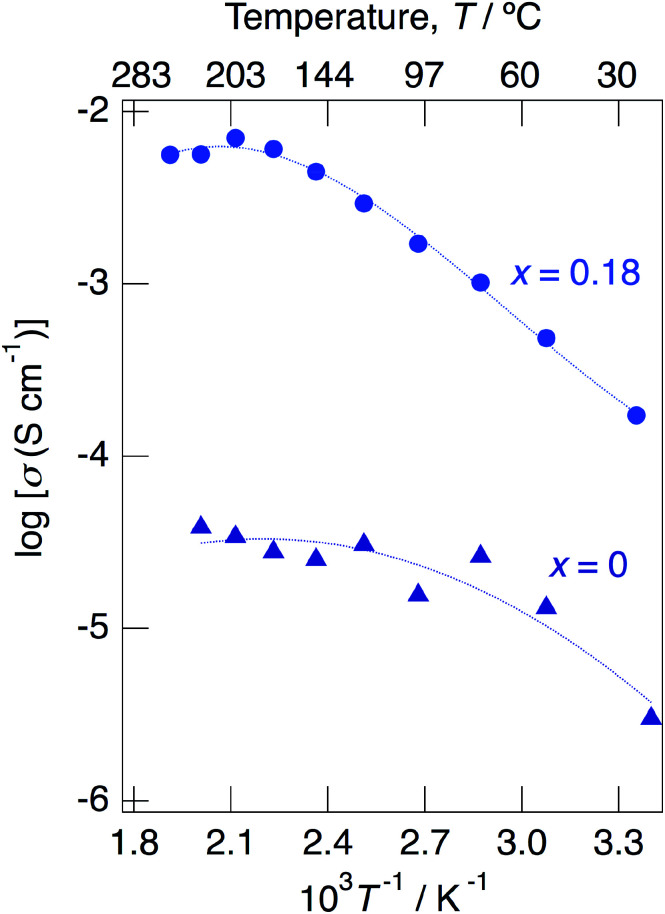
Temperature dependence of the bulk conductivity for KMg_1−*x*_H_2*x*_(PO_3_)_3_·*y*H_2_O with *x* = 0 and 0.18 in a dry Ar gas flow.

Above 52 °C, a semicircle due to the grain boundary and a spike due to the electrode contribution were observed. The bulk resistivity was obtained from the real axis intercepts of the semicircle corresponding to the grain boundary at the high frequency side. The total resistivity was obtained from the real axis intercepts of the spike corresponding to the electrode contribution. A comparison of the ionic conductivity of the sample with *x* = 0.18 measured in the closed cell and under a dry Ar gas flow is shown in Fig. S1.† The bulk and total conductivities measured in the dry Ar gas flow were lower than the total conductivity measured in the closed cell in the temperature range of 25–125 °C. The change in the conductivity with respect to humidity in the measurement atmosphere is a typical feature of proton conductors. Fig. 10 shows the temperature dependence of the bulk conductivity for KMg_1−*x*_H_2*x*_(PO_3_)_3_·*y*H_2_O in a dry Ar gas flow. The sample with *x* = 0.18 exhibited higher proton conductivity than the sample with *x* = 0 in the measured temperature range. The sample with *x* = 0.18 in KMg_1−*x*_H_2*x*_(PO_3_)_3_·*y*H_2_O exhibited high proton conductivities of 4.5 × 10^−3^ S cm^−1^ at 150 °C and 7.0 × 10^−3^ S cm^−1^ at 200 °C. The apparent activation energy in the range of 25 to 150 °C was 0.21 eV. The change of the slope from 150 °C and slight drop of the conductivity above 200 °C for KMg_1−*x*_H_2*x*_(PO_3_)_3_·*y*H_2_O with *x* = 0.18 are attributed to the loss of water of crystallization corresponding to the second dehydration step, which indicates the contribution of coordination water for the proton conduction in this temperature range. Since the high bulk conductivity was observed above 150 °C at which desorption of the adsorbed water was completed, the proton conductivity of this material must be caused by the proton diffusion in the crystal structure.

KMg_1−*x*_H_2*x*_(PO_3_)_3_·*y*H_2_O is the second tunnel phosphate that exhibits fast proton conductivity. The discovery of this material suggests that tunnel framework has tolerance for constituent cations. The combination of K^+^ and Mg^2+^ results the ordering of cations and the formation of the octahedral chain seen in Fig. 6(b). The combination of cations with the different valences and ionic radii should be one of the keys for the formation of the tunnel framework, because the PO_4_ tetrahedral chain is formed along the octahedral chain. The replacement of Rb^+^ with K^+^ changed in the space group of the unit cell with the shift of K^+^ to the off-centered position at the octahedral sites. Since the distance between K^+^ and O(3), which is the part of oxygen that form KO_6_ octahedron, is extended, K^+^ coordinates with the oxygen O(5) of the water molecule for stabilization. KO_6_ octahedron is connected to the unit composed of three PO_4_ tetrahedra by corner sharing of O(1), and MgO_6_ octahedron is connected to the unit composed of two PO_4_ tetrahedra. These PO_4_ tetrahedral units can be adjusted in length in the *z*-axis direction by changing the bond angle of P–O(1)–P for the replacement of alkali cation.

The shrinkage of the framework by the replacement of Rb^+^ with K^+^ causes two oxygen sites of water molecules in the tunnel. These water molecules exist adjacent to each other in the tunnel due to the connection to PO_4_ tetrahedral chain through protons. The distance between oxygen in water molecules adjacent in the *c*-axis direction is 1.58 Å, and that between those adjacent in the diagonally upward direction is 2.31 Å in KMg_1−*x*_H_2*x*_(PO_3_)_3_·*y*H_2_O. In RbMg_1−*x*_H_2*x*_(PO_3_)_3_·*y*H_2_O, crystallographically equivalent water molecules form chains.^17^ The distance between oxygen in adjacent water molecules in the *ab* plane is 1.80 Å, and that of water molecules adjacent each other in the *c*-direction is 2.38 Å. Due to the shrinkage of the lattice, the distances between water molecules is slightly shortened.

In KMg_1−*x*_H_2*x*_(PO_3_)_3_·*y*H_2_O, the distance of oxygen sites of water molecules is too short to occupy the adjacent O(4) and O(5) sites simultaneously. Therefore, this structural restriction would cause the cooperative movement of water molecules due to electrostatic repulsion force between these molecules above a water content of 1H_2_O per unit formula. In case the water content of KMg_1−*x*_H_2*x*_(PO_3_)_3_·*y*H_2_O is less than the maximum value of water molecules allowed by the structural restriction, which is half of the oxygen sites of water molecules, proton diffusion associated with the hopping of water molecules should be necessary. The structural refinement result suggests that the water content calculated from occupancy values of O(4) and O(5) is 1.7H_2_O per unit formula, which is less than 1/3 of all oxygen sites in the tunnel. Therefore, the distribution of water molecules would be suitable for the hopping of water molecules because it resembles the arrangement of mobile cations in other fast ionic conductors.^24–27^ Water molecules would be promoted, so as to be pushed out by the interaction between water molecules. Excess protons introduced at H(2) and H(3) sites become carriers and would diffuse in association with water molecules as H_3_O^+^ in the tunnel due to the vehicle mechanism.^17^ Further investigations, such as structural studies by the maximum entropy method at different temperatures and nuclear magnetic resonance spectroscopy studies, should provide clear evidence of the proton conduction mechanism.

KMg_1−*x*_H_2*x*_(PO_3_)_3_·*y*H_2_O exhibits high proton conductivity, which is almost comparable to that of RbMg_1−*x*_H_2*x*_(PO_3_)_3_·*y*H_2_O,^17^ over a wide temperature range. It is thought that the formation of water chains has more influence on the proton conductivity rather than the change of the local arrangement between water molecules in the tunnel phosphates. The proton conductivity of KMg_1−*x*_H_2*x*_(PO_3_)_3_·*y*H_2_O showed a positive tendency for the introduction of excess protons. The samples with the composition above *x* = 0.18 in KMg_1−*x*_H_2*x*_(PO_3_)_3_·*y*H_2_O is expected to exhibit higher proton conductivity, however crystalline samples with these compositions were not obtained in present study.

KMg_1−*x*_H_2*x*_(PO_3_)_3_·*y*H_2_O is composed of inexpensive and abundant elements and exhibits a high proton conductivity above 10^−3^ S m^−1^ at 150 °C for long periods, which are preferable for proton solid electrolyte applications. Solid acids such as CsHSO^4^ and CsH_2_PO_4_ exhibit high proton conductivity due to the proton diffusion *via* rotation and rearrangement of poly-anions above the phase transition temperature.^28^ Since the structure of the high temperature phase formed by hydrogen bonds is thermally unstable, the temperature range at which these solid acids exhibit high proton conductivity is restricted. In contrast to these solid acids, KMg_1−*x*_H_2*x*_(PO_3_)_3_·*y*H_2_O exhibits high proton conductivity at low temperature, because the proton diffusion pathway of water chains exists in the tunnel at room temperature. The rigid framework of KMg_1−*x*_H_2*x*_(PO_3_)_3_·*y*H_2_O is an advantage for the improvement of the thermal stability, however the retention of the water of crystallization is an issue to expand the operation temperature of water-contained proton conductors at high temperature. KMg_1−*x*_H_2*x*_(PO_3_)_3_·*y*H_2_O exhibits high proton conductivity of 7.0 × 10^−3^ S cm^−1^ at 200 °C at which other proton conductors that contain water molecules become unstable.^29–31^ The coincidence between the water content calculated from the weight loss at the second dehydration step observed in the TG curve and that obtained from the occupancy value of O(5) suggests that water molecules coordinated to K^+^ are retained at high temperatures. It suggests that the electrostatic bonding between cation and oxygen of water molecules is much stronger than the connection of water molecules *via* hydrogen bonds. The connection of water molecules to PO_4_ tetrahedral chains *via* hydrogen bonds contributes to the formation of water chains that are proton diffusion pathway, whereas the coordination of water molecules to large cation of K^+^ in the face-shared octahedral chains is important for the retention of water molecules in the tunnel structure at high temperatures. The strong bonding of the coordination water is considered to be disadvantageous for the fast proton diffusion, however the formation of the water chain which is suitable for the proton diffusion associated with the jump of water molecules compensates for this disadvantage and causes high proton conductivity in the tunnel phosphate. Structural study in present study suggests that the material design by utilizing the connection of oxygen of water molecules to cation in the rigid framework, which is overlooked at the material design focusing on the hydrogen bonds, has a potential to overcome the thermal instability of proton conductors. KMg_1−*x*_H_2*x*_(PO_3_)_3_·*y*H_2_O shows that tunnel phosphates are promising as fast proton conductors, and tolerance for constituent cations of the tunnel framework suggests that other isostructural compounds could be derived. Further investigation of tunnel phosphates with unique water chains is believed to provide proton solid electrolytes that exhibit higher proton conductivity in over a wide temperature range.

## Conclusions

A new proton conductor, KMg_1−*x*_H_2*x*_(PO_3_)_3_·*y*H_2_O, was synthesized by the coprecipitation method. KMg_1−*x*_H_2*x*_(PO_3_)_3_·*y*H_2_O formed a solid solution between *x* = 0 and *x* = 0.18. The dehydration of KMg_1−*x*_H_2*x*_(PO_3_)_3_·*y*H_2_O occurred at 30 °C and 150 °C, which reflects different coordination environments of the waters of crystallization. KMg_1−*x*_H_2*x*_(PO_3_)_3_·*y*H_2_O formed a tunnel framework composed of (KO_6_) and (MgO_6_) one-dimensional chains, and PO_4_ tetrahedral chains along the *c*-direction by corner-sharing. Two oxygen sites of water molecules were detected in the one-dimensional tunnel, one of which exists as a coordination water of K^+^ sites. Water molecules are connected to PO_4_ tetrahedra by hydrogen bonds and form a chain along the *c*-axis in the tunnel. The KMg_1−*x*_H_2*x*_(PO_3_)_3_·*y*H_2_O sample with *x* = 0.18 exhibited high proton conductivities of 4.5 × 10^−3^ S cm^−1^ at 150 °C and 7.0 × 10^−3^ S cm^−1^ at 200 °C in a dry Ar gas flow and maintained the total conductivity above 10^−3^ S cm^−1^ for 60 h at 150 °C under N_2_ gas atmosphere.

## Conflicts of interest

There are no conflicts to declare.

## Supplementary Material

RA-010-D0RA00690D-s001
