# Supplementary material for: Arrangement of water molecules and high proton conductivity of tunnel structure phosphates, KMg1−xH2x(PO3)3·yH2O
Source: RSC Adv. 2020 Feb 24;10(13):7803–11. doi: 10.1039/d0ra00690d (PMC9049787; doi:10.1039/d0ra00690d)
Supplement: RA-010-D0RA00690D-s001 [file RA-010-D0RA00690D-s001.pdf]

Electronic Supplementary Material (ESI) for RSC Advances.
This journal is © The Royal Society of Chemistry 2020

Supporting information for

Arrangement of water molecules and high proton conductivity of tunnel structure phosphates, $KMg_{1-x}H_{2x}(PO_3)_3 \cdot yH_2O$

Yasuaki Matsuda, Kousei Funakoshi, Ryosuke Sebe, Genki Kobayashi, Masao Yonemura, Nobuyuki Imanishi, Daisuke Mori, Shinya Higashimoto

Table S1. Interatomic distance (Å) and bond angle (°) for $KMg_{0.82}H_{0.36}(PO_3)_3$ $1.9H_2O$.

Interatomic distance	l / Å	Bond angle	φ (°)
Mg—O(2)	2.097	O(1)^a—P—O(1)^b	97.2
Mg—O(3)	2.040	O(1)^a—P—O(2)	106.8
K—O(2)	2.881	O(1)^b–P–O(2)	109.0
K–O(3)	2.778	O(1)^a–P–O(3)	110.3
P–O(1)^a	1.601	O(1)^b–P–O(3)	106.3
P–O(1)^b	1.709	O(2)–P–O(3)	123.9
P–O(2)	1.377	H(1)–O(4)–H(2)	117.7
P–O(3)	1.520	H(3)–O(5)–H(4)	136.8
K–O(4)	3.108	P^a–O(1)^b–P^c	128.9
K–O(5)	2.363		
O(1)^b–H(1)	2.336		
O(1)^b–H(2)	1.676		
O(1)^b–H(3)	1.794		
O(1)^a–H(3)	2.393		
O(2)–H(1)	2.090		
O(2)–H(2)	2.221		
O(3)–H(2)	2.200		
O(3)^a–H(4)	2.102		
O(4)–H(1)	0.281		
O(4)–H(2)	0.598		
O(5)–H(3)	0.570		
O(5)–H(4)	0.302		

Symmetry codes: ^{a}x, y, z, ^{b}-y+2/3, x-y+1/3, z+1/3, ^{c}-x+y+1/3, -x+2/3, z+2/3.

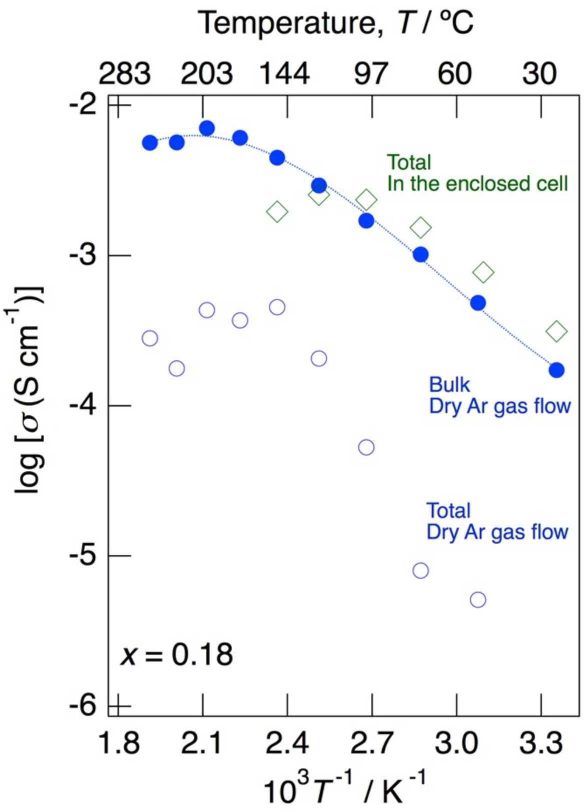

Fig. S1. Temperature dependence of the conductivity for $KMg_{1-x}H_{2x}(PO_3)_3 \cdot yH_2O$ with $x = 0.18$ measured under different atmospheres.

Proton conductivities measured in the enclosed cell tends to exhibit higher values than those measured under dry Ar gas flow. In the closed cell, since the water of crystallization desorbed from $KMg_{1-x}H_{2x}(PO_3)_3 \cdot yH_2O$ remains in the measurement atmosphere, the relative humidity of the measurement atmosphere should be increased. The water vapor should be saturated considering the amount of dehydration observed in the TG curve and the volume of the space in the cell. The total conductivity meastured in the sealed cell is equal to or higher than the bulk conductivity measured under dry Ar gas flow from 25 to 125 °C. Although the bulk conductivity measured in the sealed cell wasn’t be calculated accurately, the bulk conductivity measured in the sealed cell must be higher than that measured in the Ar gas flow. The change in the conductivity with respect to humidity in the measurement atmosphere is a phenomenon peculiar to proton conductors.
